# Evaluation of Mesoporous Silica Nanoparticles as Carriers of Triarylmethyl Radical Spin Probes for EPR Oximetry

**DOI:** 10.1021/acs.jpcb.4c06480

**Published:** 2025-01-30

**Authors:** Misa A. Shaw, Martin Poncelet, Derrick A. Banerjee, Konstantinos A. Sierros, Benoit Driesschaert

**Affiliations:** † Department of Pharmaceutical Sciences, School of Pharmacy, West Virginia University, Morgantown, West Virginia 26506, United States; ‡ In Vivo Multifunctional Magnetic Resonance Center, Robert C. Byrd Health Science Center, West Virginia University, Morgantown, West Virginia 26506, United States; § West Virginia Clinical and Translational Science Institute, West Virginia University, Morgantown, West Virginia 26506, United States; ∥ Department of Mechanical, Materials and Aerospace Engineering, Benjamin M. Statler College of Engineering and Mineral Resources, West Virginia University, Morgantown, West Virginia 26506, United States; ⊥ C. Eugene Bennett Department of Chemistry, West Virginia University, Morgantown, West Virginia 26506, United States

## Abstract

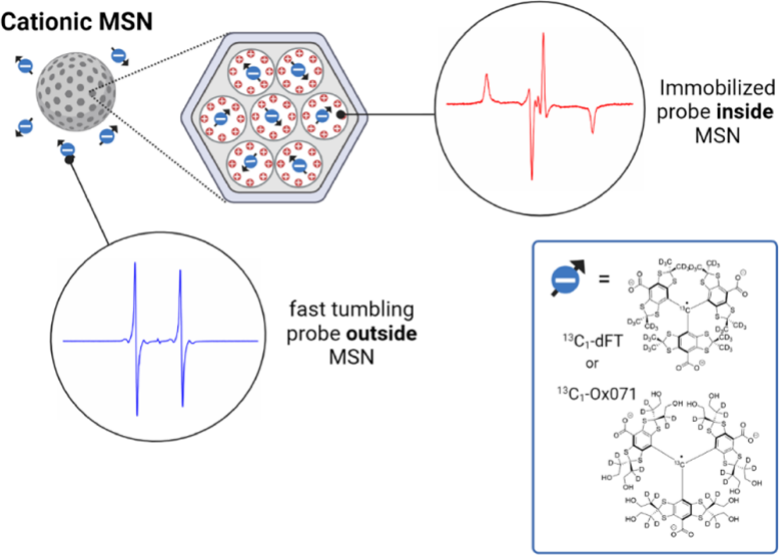

*In vivo* measurement and mapping of oxygen levels within the tissues are crucial in understanding the physiopathological processes of numerous diseases, such as cancer, diabetes, or peripheral vascular diseases. Electron paramagnetic resonance (EPR) associated with biocompatible exogenous spin probes, such as Ox071 triarylmethyl (TAM) radical, is becoming the new gold standard for oxygen mapping in preclinical settings. However, these probes do not show tissue selectivity when injected systemically, and they are not cell permeable, reporting oxygen from the extracellular compartment only. Recently, Ox071-loaded mesoporous silica nanoparticles (MSNs) were proposed for intracellular tumor oxygen mapping in both *in vitro* and *in vivo* models. However, the EPR spectrum of the Ox071 spin probe is poorly sensitive to mobility due to the small anisotropy of its g-factor and the absence of hyperfine splitting, making it more difficult to study the mobility of the radical inside the MSNs or its location. Using ^13^C_1_ isotopologues of Ox071 and the deuterated Finland trityl (dFT) spin probes, which are highly sensitive to molecular tumbling, we showed that the loading of the probes inside homemade and commercial cationic MSNs drastically decreases their mobility while the high local concentration of the probe inside the MSNs leads to dipolar line width broadening (self-relaxation). This decrease in molecular tumbling and line broadening hampers the oxygen-sensing properties of Ox071 or dFT probes used for EPR oximetry when loaded into MSNs.

## 1 Introduction

Oxygen level within the tissues and the cells is a critical biomarker of the physiology and physiopathological processes occurring in numerous diseases, such as cancer, diabetes, or peripheral vascular diseases.^1,2^ For example, hypoxia, defined as tissue *p*O_2_ < 10 mmHg, is a well-recognized hallmark of solid tumors and is responsible for deleterious consequences, including chemo- and radiotherapy resistances, metastases, or selection of cells with a more malignant phenotype, etc.^2,3^ Therefore, accurate imaging of partial pressure of oxygen (*p*O_2_) in tissue is essential in understanding disease prognosis, progression, and optimization of therapeutic intervention.

The polarographic oxygen microelectrode has been considered the gold standard for measuring *p*O_2_ in clinical settings for decades despite its severe limitations due to a lack of better techniques. Indeed, it is highly invasive, consumes the oxygen being measured, has poor sensitivity at low *p*O_2_, and provides measurement only at a single point.^4^ Magnetic resonance technologies allow for oxygen mapping in a minimally invasive way. For example, blood oxygen level-dependent (BOLD) takes advantage of the difference in magnetism between oxyhemoglobin (diamagnetic) and deoxyhemoglobin (paramagnetic), which creates an oxygen-dependent MRI contrast.^5^ However, absolute *p*O_2_ quantification remains challenging. ^19^F-MRI oximetry with perfluorocarbon provides more quantitative measurements but suffers from low sensitivity.^6^ On the other hand, low-frequency electron paramagnetic resonance (EPR) associated with exogenous oxygen-sensitive molecular probes is a minimally invasive emerging technique that can directly provide quantitative *p*O_2_*in vivo* and is currently assessed in cancer patients. Current clinical trials use a small oxygen sensor (0.6 × 5 mm) named OxyChip, composed of an oxygen-sensitive particulate lithium octa-butoxynaphthalocyanine (LiNc-BuO) embedded into biocompatible FDA-approved oxygen-permeable polydimethylsiloxane (PDMS) polymer. The OxyChip is implanted at the site of the desired oxygen measurement.^7,8^ A limitation is that OxyChip can only measure *p*O_2_ in the vicinity of the sensor,^9^ making it unsuitable for imaging.^10^

**Figure 1:**
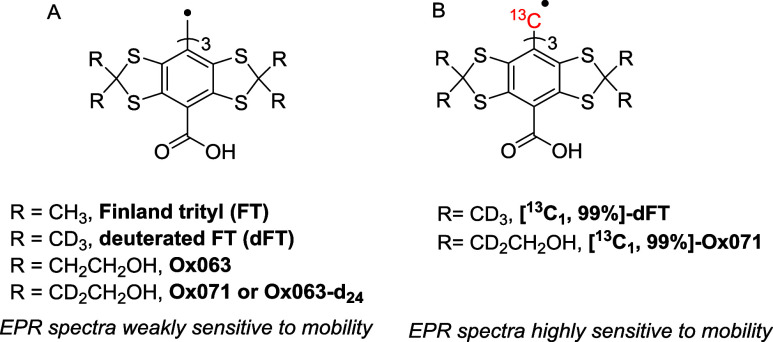
(A) Structures of TAMs weakly sensitive to mobility used for EPR oximetry and (B) ^13^C_1_-labeled TAM highly sensitive to mobility.

Alternatively, water-soluble triarylmethyl (TAM, trityl) radical probes, including the Finland Trityl (FT), Ox063, and their partially deuterated analogs dFT, Ox063-*d*_24_ (also named Ox071) (Figure 1A), are optimal for EPR oxygen imaging due to their tissue diffusion, extraordinary biostability, high biocompatibility *in vivo* (LD_50_ (Ox063/71) = 8 mmol/kg in mice)^11^ and narrow line widths (long relaxation times),^12^ leading to high spatial and functional resolutions. With these unprecedented features, creative research in TAM design has led to EPR probes with sensitivities to multiple relevant biological parameters, including *p*O_2_,^13^ pH,^14,15^ inorganic phosphate concentrations,^16^ thiol concentrations,^17,18^ redox,^19−21^ microviscosity^22−24^ and enzyme activity.^25^ Despite the advantage of soluble TAM probes over their particulate counterparts, no TAM probe has yet received regulatory clearance for human use.

Moreover, because of their charged nature and large size, TAM radicals are not cell permeable, reporting physiological parameters only from the extracellular compartment. Acetoxymethoxycarbonyl,^26^ poly arginine^27^ TAM derivatives and liposome-based formulations^28^ have been proposed for intracellular delivery of TAMs but have yet to show efficacy *in vivo*. More recently, Chen and colleagues reported Ox071-loaded fluorescent mesoporous silica nanoparticles (FMSNs) for intracellular tumor EPR oximetric imaging and demonstrated their applications in a mouse model of colon cancer.^29^ Bioapplication of MSNs (*i.e*., MCM-41) has been a popular method for nanomedicine and drug delivery since the 1990s because of their highly manipulable and customizable features, such as nanoparticle size, pore size, charges, and surface.^30,31^ It is also known for its extensive surface area, which allows for increased analyte loading.^32^

Nitroxide radicals are another type of soluble EPR probes. Because of their high spectral sensitivity to mobility, nitroxide radical spin probes have been utilized extensively to study the interaction of spin probes with MSNs. It has been shown that depending on the particular structure of the nitroxide and the type of MSNs, the radical can experience fast tumbling or have its mobility restricted.^33^ TAM radicals (FT/dFT or Ox063/71) show higher stability *in vivo* and longer relaxation times, making them superior for *in vivo* EPR applications to nitroxides. Thus, MSNs loaded with TAM spin probes are attractive as they would enable tissue targeting, intracellular delivery, dual imaging modality (*e.g.*, EPR and fluorescence (FMSNs)), or co-loading of EPR probes with therapeutic agents (theragnostic). Importantly, the local TAM probe concentrations can be controlled inside MSNs. Indeed, high probe concentrations can act as a confounding factor to oxygen (self-relaxation).

Nevertheless, TAM radicals have a small *g*-factor anisotropy, and FT/dFT or Ox063/71 were designed to eliminate all hyperfine splittings to optimize for oximetric applications. Therefore, those radicals are poorly sensitive to mobility. Still, a change in tumbling rates could modulate the relaxation times T_1e_ and T_2e_ and the EPR line width, which are the reporting parameters used for oxygen measurement.

In Chen’s FMSNs study, the negatively charged Ox071 oxygen probe was loaded into cationic MSNs. The spectrum of Ox071 encapsulated in FMSNs remained the same as the TAM radical alone in solution with no spectral broadening due to decreased mobility or concentration-induced self-broadening. However, as mentioned earlier, assessing the mobility and location of Ox071 is challenging. We recently reported isotopologues of dFT and Ox071 labeled ^**13**^C at the central carbon (^**13**^C_**1**_-dFT and ^**13**^C_**1**_-Ox071, Figure 1B).^23,24^ The large anisotropy of the hyperfine interaction with the ^**13**^C_**1**_ (A_*x*_ = A_*y*_ = 18 ± 2 MHz, A_*z*_ = 160 ± 5 MHz) makes those radicals very sensitive to rotational diffusion, making them ideal candidates to assess the mobility of TAMs inside MSNs. Hereby, we report the effect of loading dFT or Ox071 into MSNs on their mobility. We first synthesized MSNs with *n*-octane (hMSNs) to enlarge the pore sizes as previously described.^29^ We then observed the molecular tumbling and concentration-induced line broadening of the TAM radicals mixed at various ratios with hMSNs. We verified the location of the probe and compared hMSN to commercially available MSNs.

## 2 Experimental Section

### 2.1 Reagents and Materials

The following list of chemicals was used in the synthesis of mesoporous silica nanoparticles: hexadecyltrimethylammonium bromide (CTAB, 99%), ammonium hydroxide (30–33%), tetraethyl orthosilicate (TEOS, 98%), (3-Aminopropyl) triethoxysilane (APTES, 99%), and ammonium nitrate (≥98%) were purchased from Sigma-Aldrich (St. Louis, MO). *n*-Octane (98+%), *N*-[3-(Trimethoxysilyl)propyl]-*N*,*N*,*N*-trimethylammonium chloride in 50% methanol, methanol, and ethanol absolute (200 Proof) were purchased from Thermo Fisher Scientific (Waltham, MA). Commercially available aminated (cationic) MSNs were purchased from NanoComposix, Inc. (pMSNs: #SHSD100, Lot #SAM-0388-SAM0390, San Diego, CA). ^13^C_1_-dFT,^23^^13^C_1_-OX071,^24^ dFT,^34^ and Ox071^35^ radicals in their sodium carboxylate form were synthesized as previously reported. For transmission electron microscopy (TEM), carbon Type-B, 300 mesh, copper TEM grid was purchased from Ted Pella, Inc. (Redding, CA).

### 2.2 Synthesis of Mesoporous Silica Nanoparticles, MSNs

MSNs were synthesized using the Stöber process with some modifications.^29,36^ Briefly, 0.58 g of hexadecyltrimethylammonium bromide (CTAB) was dissolved in 300 mL of 0.17 M ammonium hydroxide solution, and 7.11 mL of *n*-octane was added. The mixture was stirred at 40 °C for 1 h. Then, 5 mL of 0.1 M APTES and 5 mL of 0.2 M TEOS were added to the mixture and stirred vigorously for 4 h at 40 °C. 5 mL of 1.0 M TEOS was added to the mixture drop-by-drop while stirring the mixture vigorously at 40 °C for 1 h. The mixture was stirred at 40 °C for 20–24 h. The next day, the mixture was washed with deionized (DI) water and centrifuged at 21,000 RCF for 30 min (3X). The pellet was then resuspended in 50 mL of 200-proof ethanol solution with 250 mg ammonium nitrate and kept overnight at room temperature (RT). The mixture was then gently stirred at 60 °C for 2 h and centrifuged at 21,000 RCF for 30 min to remove CTAB from the hMSNs. The same stirring procedure was repeated to ensure the complete removal of CTAB from the hMSNs. The pellet was then resuspended with 50 mL methanol containing 300 μL of *N*-trimethoxysilylpropyl-*N*,*N*,*N*-trimethylammonium chloride (TA) and stirred with a condenser overnight at 60 °C. The hMSN pellets were washed with 200-proof ethanol and centrifuged at 21,000 RCF for 30 min (3×) the next day. Lastly, the final hMSNs powder was produced by evaporating the ethanol using a rotary evaporator and dried under a high vacuum.

### 2.3 General Characterization

The particle size of the hMSNs was determined using a JEOL JEM-2100 Transmission Electron Microscope (Peabody, MA) and ImageJ. The size was averaged over ten nanoparticles. The Zeta potential was measured using a Malvern Zetasizer Nano Z (Malvern, UK). Zeta potential titration was performed by adjusting the pH of the hMSNs in DI water (2 mg/mL) with NaOH or HCl (<3% dilution) (Figure S1A). Porosity and pore size were determined using a Micromeritics ASAP 2020 analyzer (Norcross, GA). EPR spectra were recorded on a Bruker Elexsys E580 X-band spectrometer (Billerica, MA) at room temperature under normal air conditions (21% O_2_). General instrumental settings for the ^13^C_1_ TAMs were as follows unless otherwise mentioned: microwave power 4.74 mW, sweep width 100 G, modulation frequency 100 kHz, modulation amplitude 0.50 or 1.50 G, time constant 40.96 ms, conversion time 40.96 ms, sweep time 83.89 s, and spectral points 2048. For the natural abundance probes in C_1_ (dFT and Ox071), the instrument settings were as follows: microwave power 0.47 mW, sweep width 5 G, modulation frequency 30 kHz, modulation amplitude 0.05 G, time constant 40.96 ms, conversion time 40.96 ms, and spectral points 1024, EPR measurements were performed in a 50 μL glass capillary tube. EPR spectra fitting and simulation were performed using the EasySpin 6 package for MATLAB (ver 2022b).^37^

### 2.4 Molecular Tumbling of TAM Radicals in MSNs

Aminated hMSNs (5 mg) were resuspended with 0.2 mL of TAM radicals (^13^C_1_-dFT or ^13^C_1_-Ox071) at different concentrations: 0.25 mM (0.05 μmol), 0.5 mM (0.1 μmol), 1 mM (0.2 μmol), 2 mM (0.4 μmol), and 4 mM (0.8 μmol) and the pH of each sample was adjusted to ∼7.0 using NaOH or HCl (<3% dilution) solutions prior to starting the incubation at room temperature (RT) for 30 min using an Eppendorf thermomixer (Enfield, CT) at 300 rpm. Samples were then transferred to a glass capillary (50 μL) for recording EPR spectra at RT under air.

In addition, samples with dFT and Ox071 natural abundance of the central carbon (C_1_) were prepared at 0.05 μmol (0.25 mM), with and without 5 mg of homemade and purchased MSNs.

### 2.5 Verification of TAM Location

Samples at 0.25 mM (0.05 μmol) ^13^C_1_-dFT or ^13^C_1_-Ox071 in 5 mg hMSNs at pH ∼ 7.0, which showed fast tumbling peaks on the EPR spectrum, were centrifuged at 13,500 RCF at 15 °C for 15 min to separate supernatant and pellets. The pellet was resuspended with 0.2 mL DI water, and EPR spectra of the supernatant and the resuspended pellet samples were recorded to verify the location of the TAM radicals (*i.e.*, inside or outside the hMSNs).

### 2.6 Comparison of Homemade (hMSN) with Purchased (pMSN)

hMSNs or purchased cationic MSNs (pMSNs, 5 mg) were resuspended with 0.2 mL of 0.05 μmol (0.25 mM) TAM radicals (^13^C_1_-dFT or ^13^C_1_-Ox071), and the pH of the solution was adjusted to ∼7.0. Samples were incubated at RT for 30 min using a thermomixer at 300 rpm and then transferred to a glass capillary (50 μL) for recording EPR spectra at RT under air.

### 2.7 Loading Capacity and Release

Aminated hMSNs (5 mg) were resuspended with 0.2 mL of TAM radicals (^13^C_1_-dFT or ^13^C_1_-Ox071) at 10 mM in a microcentrifuge tube, and the pH was adjusted to ∼7.0 before incubation at room temperature (RT) for 30 min using an Eppendorf thermomixer (Enfield, CT) at 300 rpm. The tube was centrifuged at 13,500 RCF at 15 °C for 15 min, and the supernatant was separated. The concentration of TAM in the supernatant was measured by UV–vis. The pellet was washed two more times with 0.2 mL DI water, and the TAM concentration was measured in both supernatants using UV–vis. The total amount of probe recovered in the three supernatants was subtracted from the initial amount to calculate the loading capacity using the following formula: Loading capacity = (mass of TAM inside MSNs/mass of MSNs) × 100. The loading capacity reached 20 ± 5% for ^13^C_1_-dFT and 21 ± 5% for ^13^C_1_-Ox071. The loading efficiency was calculated as the ratio of the mass of TAM inside MSN with the total mass of TAM added × 100. The loading efficiency reached 36 ± 5% for ^13^C_1_-dFT and 44% for ^13^C_1_-Ox071. Then, the pellets were resuspended in 0.2 mL of DI water pH ∼ 7 and transferred to a glass capillary (50 μL), and the EPR spectra were recorded immediately and after 24 h at RT under air. In addition, EPR spectra of the pellets resuspended in DI water pH ∼ 7, containing 150 mM of NaCl, were also recorded.

## 3 Results and Discussion

### 3.1 Synthesis and Characterization of MSNs

The hMSNs were synthesized as previously described with minor modifications.^29,36^ Once the surfactant was removed, hMSNs were further aminated by incubating with *N*-trimethoxysilylpropyl-*N*,*N*,*N*-trimethylammonium chloride (TA)/MeOH solution (0.6% v/v) under reflux overnight at 60 °C. The pore size, surface area, and pore volume were determined by Brunauer–Emmett–Teller (BET) analysis and are summarized in Table 1 and compared to commercially available MSNs (pMSNs). As expected with the use of *n*-octane, the pore size of hMSNs is larger than that of pMSNs.^29^ The zeta potential was measured for pH values from 4 to 9.5 (Figure S1A). hMSNs have a zeta potential of 14.0 mV at neutral pH (Table 1), and an isoelectric point at pH = 7.4. hMSNs are cationic for lower pH and anionic for higher pH. This can be rationalized as hMSNs possess both primary amines from the aminopropyl moiety (pH-sensitive) and quaternary ammoniums from the propyl-*N,N,N*-trimethylammonium moiety (pH-insensitive) but also silanol functions. When the pH is lower than the p*K*_a_ of the aminopropyl moiety (p*K*_a_ ∼ 7.5), all the nitrogens are cationic, while the silanol groups are neutral, resulting in positive zeta potential. For higher pH values, the primary amines become neutral while the silanol becomes negatively charged, resulting in a negative zeta potential when the amount of negatively charged silanol becomes superior to the number of quaternary amines. pMSNs showed a similar zeta potential of +13.5 mV at neutral pH. The diameter of the hMSNs was determined to be 80 ± 9 nm from the TEM images (Figure S1B), comparable with the pMSNs, which have a diameter of 89 ± 14 nm (from the manufacturer’s certificate of analysis).

**Table 1:** Characteristics of MSNs

Sample Name	Pore Diameter (nm)	Surface Area (m^2^/g_particles_)	Pore Volume (cm^3^/g)	Zeta Potential pH = 7 (mV)	Particle Size (nm)
Homemade MSNs (hMSNs)	10.6 ± 1.7	319.1 ± 2.6	1.3 ± 0.2	+14.0 ± 1.0	80 ± 9
Purchased MSNs (pMSNs)	3.18^a^	655	0.6	+13.5 ± 1.0	89 ± 14^a^

a Information is based on the Certificate of Analysis (NanoComposix: SHAD-100-25MG; Lot # SAM-0388-SAM0390).

### 3.2 Molecular Tumbling of TAM Radicals in MSNs

**Figure 2:**
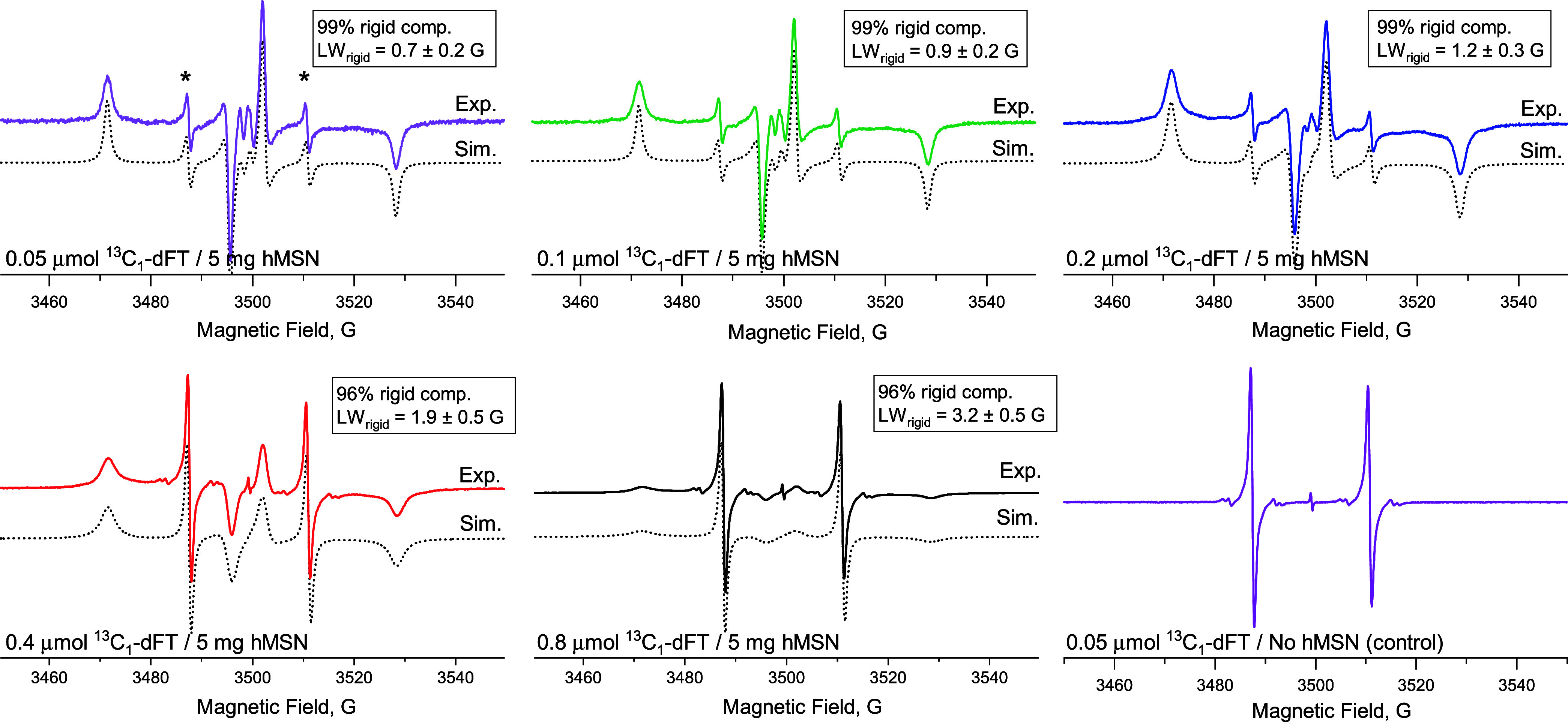
X-Band EPR of 5 mg of hMSNs with various quantities of ^13^C_1_-dFT radical in 0.2 mL: 0.05 μmol (0.25 mM, purple), 0.1 μmol (0.5 mM, green), 0.2 μmol (1 mM, blue), 0.4 μmol (2 mM, red), 0.8 μmol (4 mM, black), and 4 mM of ^13^C_1_-dFT without MSNs (pink) at pH = 7. The first spectrum was simulated (dashed gray) with the “chili” function of EasySpin with the following parameters: g_*x*_ = 2.0032, g_*y*_ = 2.0031, g_*z*_ = 2.00265, A_*x*_ = 19 MHz, A_*y*_ = 19 MHz, A_*z*_ = 160 MHz with two spectral components. The first with a correlation time τ_r_ = 0.1 ms (to simulate the rigid limit) and the second with τ_r_ = 0.26 ns (fast tumbling). The percentage and the Lorentz line width of the rigid component determined by simulation are indicated on the spectra. (*) shows the fast-tumbling component on the first spectrum only.

First, we assessed the effect of TAM loading into hMSNs on the mobility of the probe with the amphiphilic ^13^C_1_-dFT. 0.2 mL of various concentrations of radicals (0.25 to 4 mM; 0.05 μmol to 0.8 μmol TAM) were mixed with 5 mg of hMSNs at pH = 7. As shown in Figure 2, at the lowest concentration of TAM (0.05 μmol, 0.25 mM), the spectrum exhibits two components: a rigid spectral component, indicating that the radical is in interaction with the hMSNs, and this interaction leads to a drastic decrease in the tumbling rate of the probe and a fast-tumbling component (doublet pattern labeled with * on the spectrum). The peak-to-peak line widths of the doublets (∼0.7 G) are similar to the spectrum recorded for the probe in the absence of MSNs, suggesting that this spectral feature could correspond to the probe outside the MSNs. The first spectrum can be simulated using EasySpin with 1% of the fast tumbling component with a correlation time of 0.26 ns in addition to the rigid component. 0.05 μmol of ^13^C_1_-dFT in 5 mg of hMSNs with a pore volume of 1.3 cm^3^/g leads to a concentration of TAM inside the hMSNs of ∼7.5 mM. When the concentration of the spin probe increases, the ratio of intensity between the fast-tumbling and the rigid component increases, suggesting at first look a higher proportion of probes outside the MSNs.

**Figure 3:**
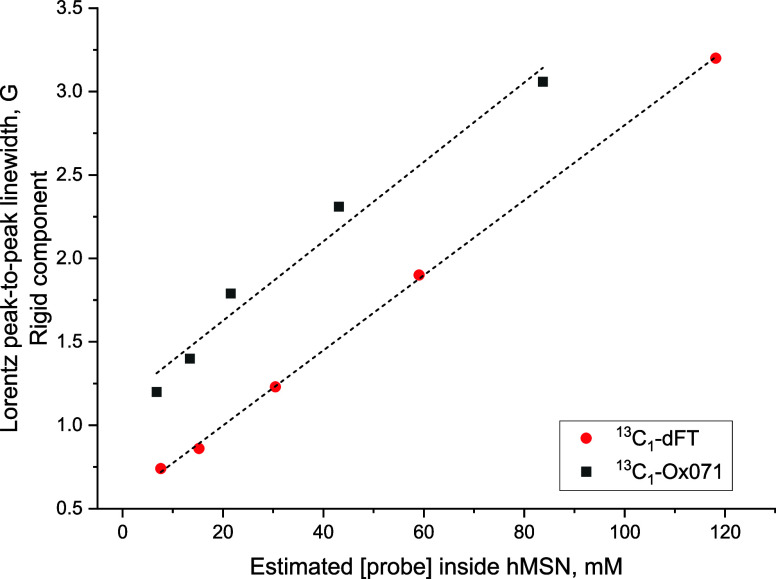
Lorentz peak-to-peak line widths for the rigid component as a function of the probe concentration inside the hMSNs. The Lorentz peak-to-peak was determined by spectral simulations (Figures 2 and 4). The concentration of the probe inside the MSNs was estimated using the total amount added (0.05–0.8 μmol in 5 mg of hMSNs with a pore volume of 1.3 cm^3^/g). The concentration was corrected for the fraction of probe inside MSNs as determined by spectral simulations and indicated in Figures 2 and 4.

However, spectral simulations show an increase of the fast tumbling component from 1% to 4% only, indicating that most of the probe is inside the hMSNs, event at 0.8 μmol of ^13^C_1_-dFT mixed with 5 mg of MSNs, corresponding to a concentration of ^13^C_1_-dFT inside MSNs of ∼120 mM. This high concentration leads to a large broadening of the EPR line width of the rigid component through dipolar interaction (self-relaxation) from 0.7 ± 0.2 G to 3.2 ± 0.5 G (Figure 3). On the other hand, the line widths of the fast-tumbling component remain constant.

**Figure 4:**
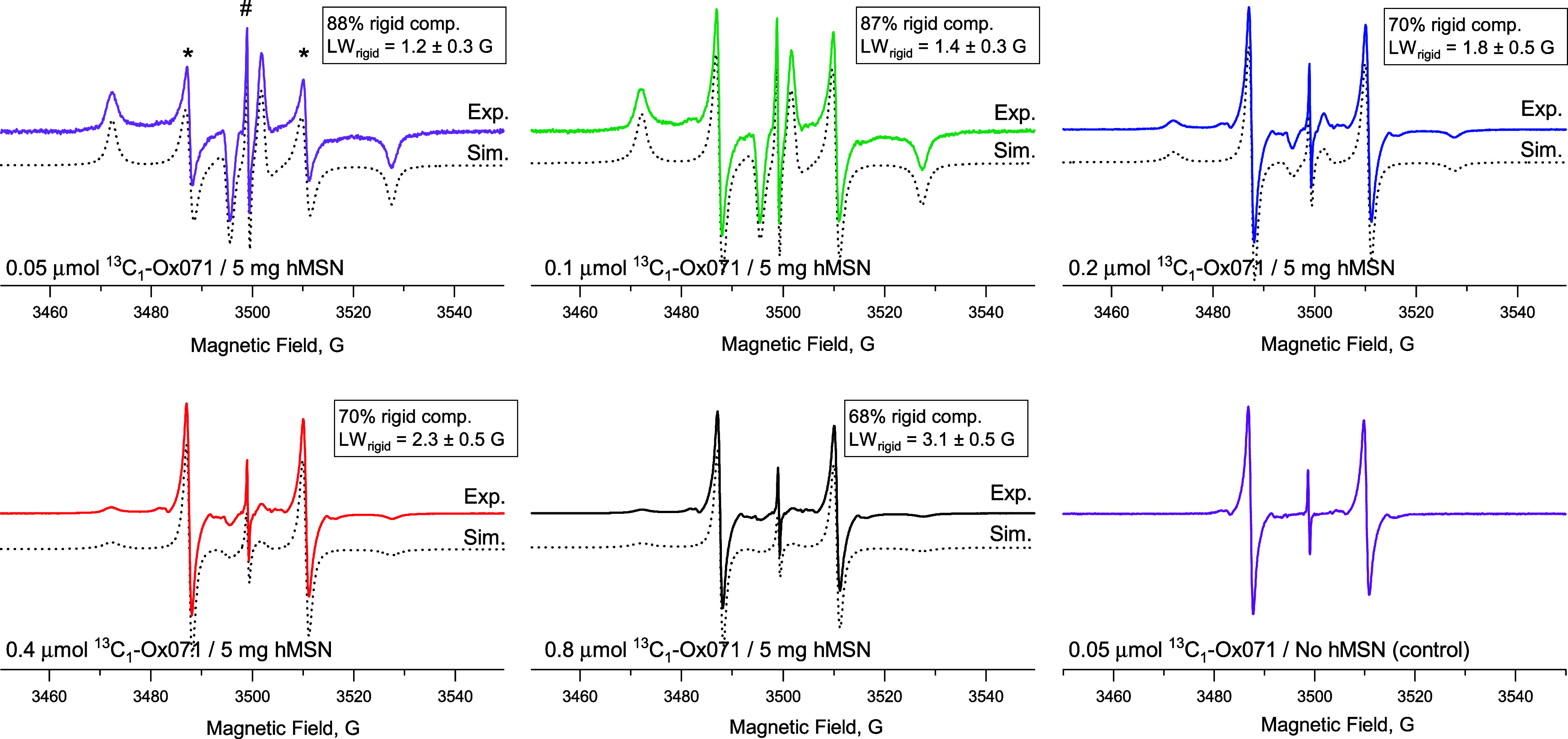
X-Band EPR of 5 mg of hMSNs with various quantities of ^13^C_1_-Ox071 radical in 0.2 mL: 0.05 μmol (0.25 mM, purple), 0.1 μmol (0.5 mM, green), 0.2 μmol (1 mM, blue), 0.4 μmol (2 mM, red), 0.8 μmol (4 mM, black), and 4 mM of ^13^C_1_-Ox071 without MSNs (pink) at pH = 7. The first spectrum was simulated (dashed gray) with the “chili” function of EasySpin with the following parameters: component 1; g_*x*_ = 2.00335, g_*y*_ = 2.00315, g_*z*_ = 2.00262, A_*x*_ = 19 MHz, A_*y*_ = 19 MHz, A_*z*_ = 155 MHz, τ_r_ = 0.1 ms (to simulate the rigid limit), component 2; g_*x*_ = 2.00335, g_*y*_ = 2.00315, g_*z*_ = 2.00262, A_*x*_ = 19 MHz, A_*y*_ = 19 MHz, A_*z*_ = 155 MHz, τ_r_ = 0.48 ns, component 3; g_*x*_ = 2.00335, g_*y*_ = 2.00315, g_*z*_ = 2.00262, τ_r_ = 0.48 ns, weight: 1%. (*) shows the fast tumbling component, while (#) shows the 1% ^13^C_1_-Ox071, on the first spectrum only.

The same experiments were performed with the hydrophilic ^13^C_1_-Ox071 (Figure 4) with similar results. At the lowest loading, the doublet corresponding to the faster tumbling component (labeled * on the spectrum) is also visible. The peak-to-peak line width of the doublet (0.9 G) is consistent with the spectrum recorded in the absence of MSNs and could be attributed to the probe outside the MSNs. The spectra can be simulated with 11% of the fast-tumbling component. Overall, the results with increased concentration of spin probe follow the same trend as with the ^13^C_1_-dFT, but with a fraction of rigid component decreasing by 20% from 0.05 to 0.8 μmol of ^13^C_1_-Ox071 with 5 mg of hMSN. In addition, a single-line spectrum corresponding to the 1% residual ^12^C_1_-Ox071 of the fast-tumbling component (labeled # on the spectrum) was also visible on all spectra, while it was less visible in the case of ^13^C_1_-dFT (Figure 2). This is because the line widths of the ^13^C_1_-Ox071 are much larger than those of the ^13^C_1_-dFT under the same conditions because of the slower tumbling rate resulting from its larger size. The narrow line of the ^12^C_1_ probe is, therefore, more intense. Moreover, as the loading of the hMSN increases, the peak-to-peak Lorentz EPR line width of the rigid component increases as a result of dipolar interaction (self-relaxation) from 1.2 ± 0.3 G to 3.1 ± 0.5 G (Figure 3) while line widths of the fast-tumbling component remain constant. The concentration of ^13^C_1_-Ox071 inside hMSNs was estimated to increase from 7 mM for 0.05 μmol of the probe mixed with 5 mg hMSN to 83 mM for 0.8 μmol of ^13^C_1_-Ox071 (Figure 3).

**Figure 5:**
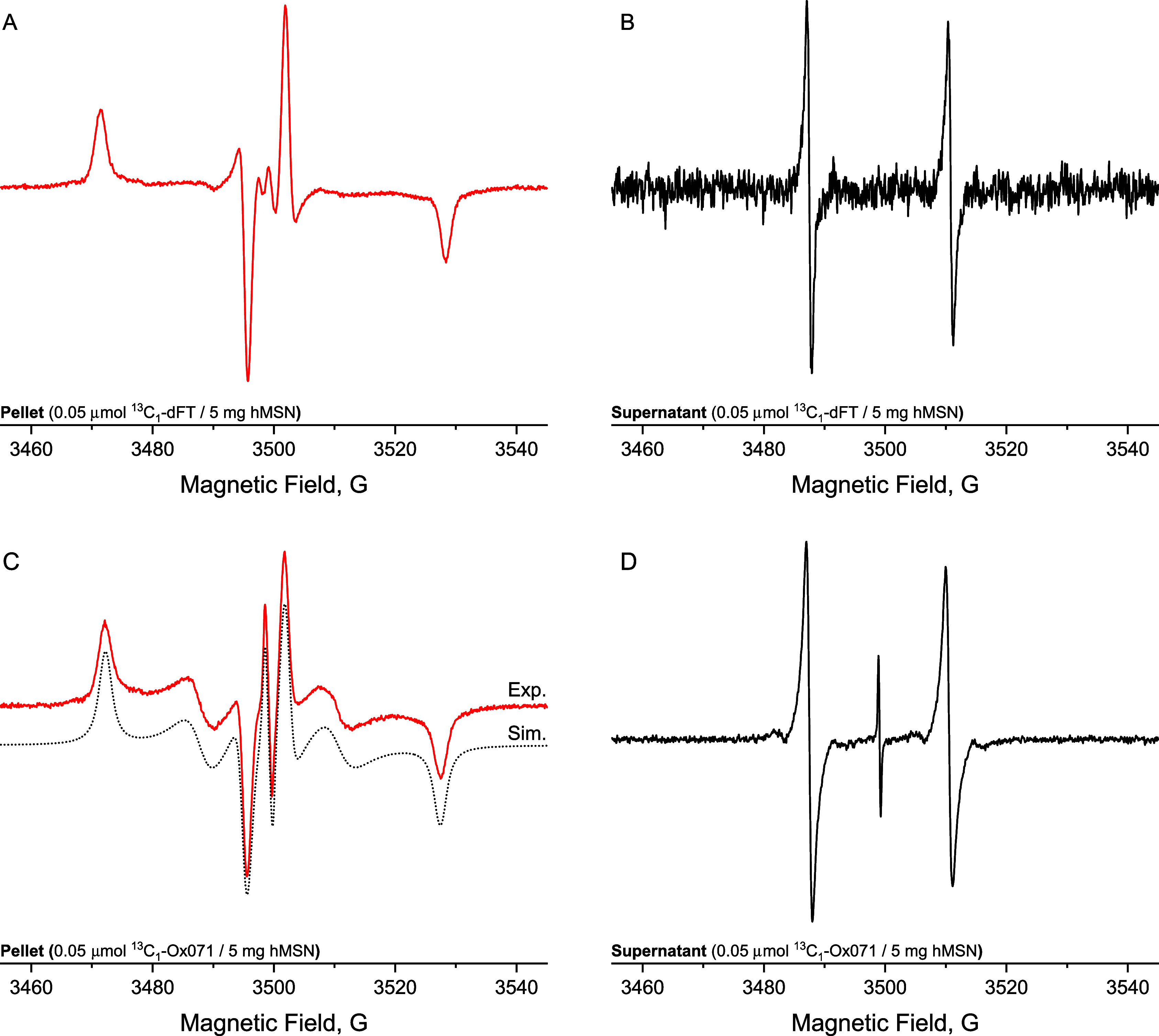
X-Band EPR spectra of pellet and supernatant of TAM-loaded hMSNs. 5 mg hMSNs and 0.05 μmol TAM in 0.2 mL DI water (pH = 7) were centrifuged, the pellet and supernatant were separated, and the X-Band spectra were recorded. (A) ^13^C_1_-dFT/hMSNs pellet resuspended in 0.2 mL of DI water and (B) supernatant. (C) ^13^C_1_-Ox071/hMSNs pellet resuspended in 0.2 mL of DI water and (D) supernatant, the pellet EPR spectrum of the pellet was simulated (dashed gray) with the “chili” function of EasySpin with the following parameters: component 1; g_*x*_ = 2.00335, g_*y*_ = 2.00315, g_*z*_ = 2.00262, A_*x*_ = 19 MHz, A_*y*_ = 19 MHz, A_*z*_ = 155 MHz, τ_r_ = 0.1 ms (to simulate the rigid limit), weight: 83%, component 2; g_*x*_ = 2.00335, g_*y*_ = 2.00315, g_*z*_ = 2.00262, A_*x*_ = 19 MHz, A_*y*_ = 19 MHz, A_*z*_ = 155 MHz, τ_r_ = 2.5 ns, weight: 16%, component 3; g_*x*_ = 2.00335, g_*y*_ = 2.00315, g_*z*_ = 2.00262, τ_r_ = 0.45 ns, weight: 1%.

In order to verify that the fast-tumbling spectral components observed for ^13^C_1_-dFT/hMSNs and ^13^C_1_-Ox071/hMSNs arise from the probe localized outside the hMSNs, samples (0.05 μmol TAM per 5 mg hMSNs) that exhibited both rigid and fast-tumbling components on the EPR spectra were centrifuged to separate the hMSNs from the solution. The EPR spectra of the supernatant and the pellet resuspended in 0.2 mL of DI water were recorded (Figure 5). In the case of ^13^C_1_-dFT, the pellet contained only the immobilized probe. On the other hand, the supernatant showed the spectrum corresponding to the probe in fast-tumbling (peak-to-peak line width: 0.7 G, τ_r_ = 0.26 ns), confirming the assignment of this component to the probe outside MSNs. Interestingly, the results were slightly different for the ^13^C_1_-Ox071 hMSNs samples. While the EPR spectrum of the supernatant showed the fast-tumbling component as seen in ^13^C_1_-dFT, the spectrum from the pellet showed the expected immobilized spectrum but also a doublet that exhibited broader line width (∼5 G) than the doublet obtained in the supernatant. Multiple washes of the pellet with DI water did not lead to a decrease in this spectral feature. The spectrum of the pellet was simulated with 15% of ^13^C_1_-Ox071 with a tumbling correlation time of 2.5 ns, suggesting that the component is assigned to the probe interacting with the MSNs but is not fully immobilized.

**Figure 6:**
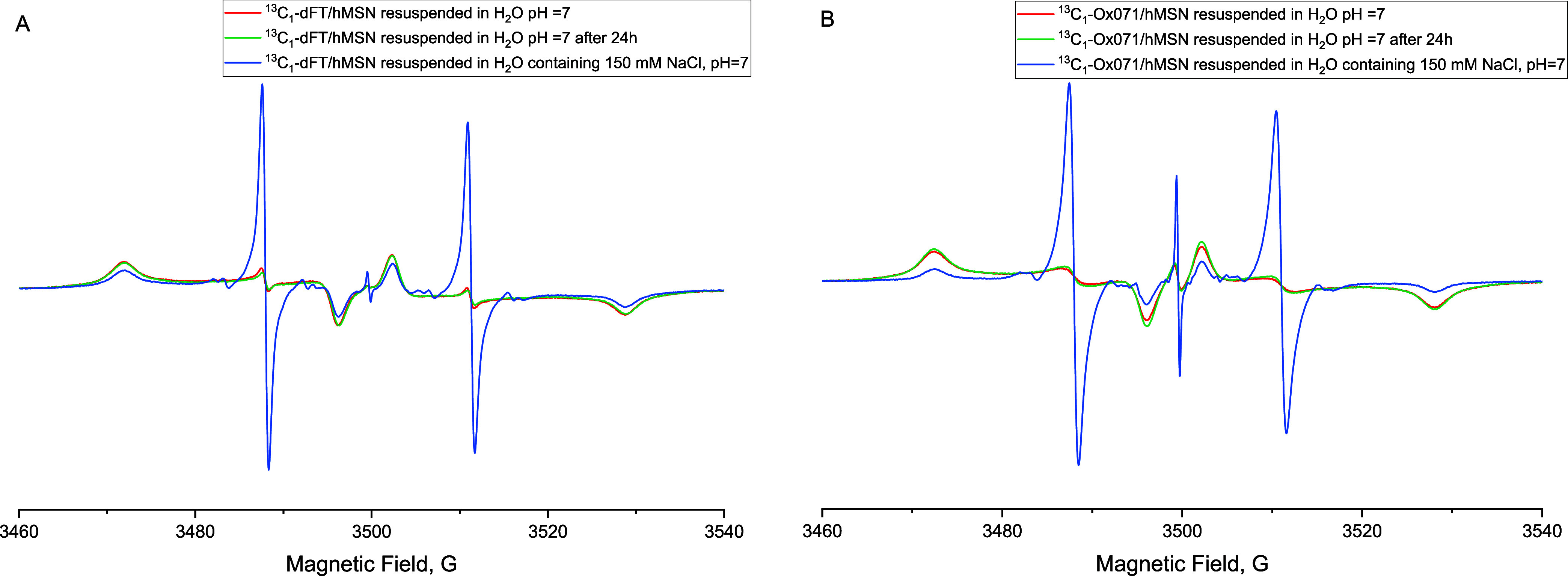
X-Band spectra of hMSNs saturated with ^13^C_1_-dFT (A) or Ox071 (B) resuspended in DI water at pH ∼ 7 with or without NaCl 150 mM.

Next, the loading capacity and release of ^13^C_1_-dFT/Ox071 from hMSNs were assessed by mixing 5 mg of hMSNs with 0.2 mL of 10 mM ^13^C_1_-TAMs in water at pH ∼ 7. After 30 min of incubation, the TAM-loaded MSNs were collected by ultracentrifugation and washed two times with DI water. The loading capacity was calculated from the amount of TAMs in the supernatants measured by UV–vis and reached 20 ± 5% and 21 ± 5% for ^13^C_1_-dFT and ^13^C_1_-Ox071, respectively. In addition, the pellets were resuspended in DI water at pH ∼ 7, and the EPR spectra were recorded immediately and after 24 h. As shown in Figure 6, no significant amount of ^13^C_1_-dFT/Ox071 was released from the hMSNs after 24 h. However, when the TAM-loaded hMSNs were resuspended in water containing 150 mM of NaCl, the fast tumbling component immediately increased, showing that the loading of ^13^C_1_-dFT/Ox071 is reversible and can be displaced by other anions.

**Figure 7:**
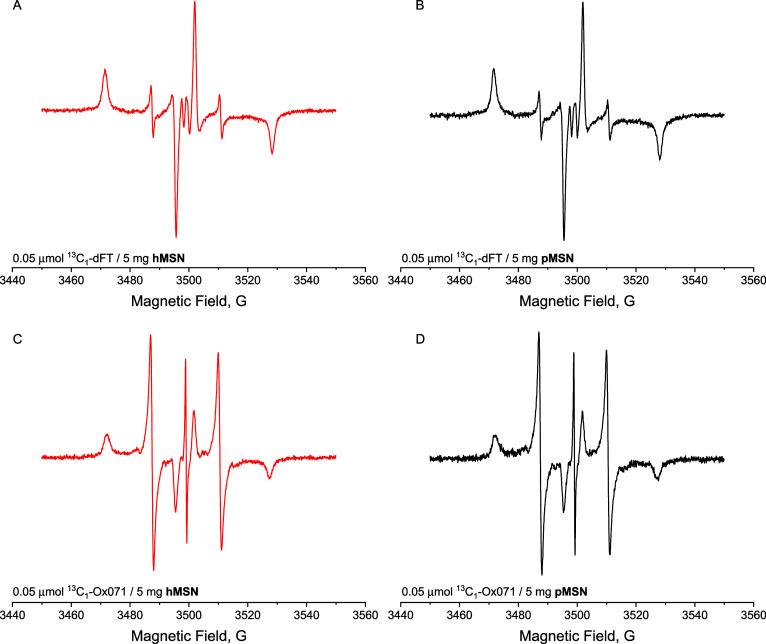
X-Band EPR spectra of 5 mg MSNs and 0.05 μmol TAM in 0.2 mL DI water (pH = 7). (A) ^13^C_1_-dFT with hMSNs, (B) ^13^C_1_-dFT with pMSNs, (C) ^13^C_1_-Ox071 with hMSNs, and (D) ^13^C_1_-Ox071 with pMSNs.

Then, we compared our homemade MSNs (hMSNs) to commercially available aminated MSNs (pMSNs) for 0.05 μmol TAM per 5 mg hMSNs. As shown in Figure 7, both hMSNs and pMSNs show similar results for both probes with immobilization inside the MSNs and fast tumbling outside.

**Figure 8:**
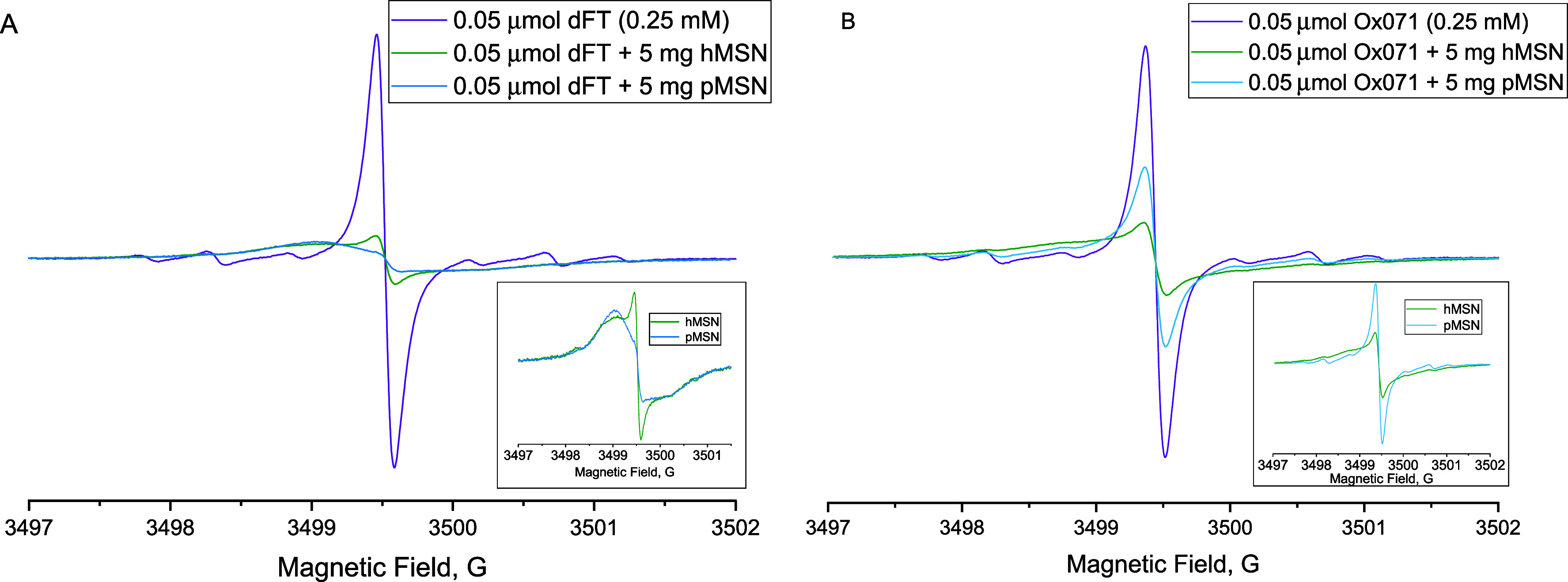
EPR spectra of 0.05 μmol of dFT (A) and Ox071 (B) in 0.2 mL of DI water at pH ∼ 7 mixed with 5 mg of hMSNs.

Our results on ^13^C_1_-dFT/Ox071 loaded into hMSNs suggest that the restricted mobility and dipolar broadening would impair the oxygen-sensing properties of the non-^13^C_1_-labeled probes. Therefore, C_1_ natural abundance dFT and Ox071 were mixed with hMSNs (0.05 μmol TAM per 5 mg hMSNs), and the EPR spectra were recorded. As Figure 8 shows, the addition of MSNs to both dFT and Ox071 solutions leads to a decrease in signal intensity and the appearance of a broad, slightly asymmetric signal, which overlaps with the narrower peak of the probe outside the nanoparticle. The broad peak is the consequence of the probe inside the MSNs with restricted mobility and concentration-induced dipolar broadening. It is worth mentioning that this concentration of TAMs is ∼50-fold lower than the Ox071 concentration used in the previous report.^29^ A higher loading will result in stronger self-broadening inside the nanoparticles.

## 4 Conclusion

Cationic mesoporous silica nanoparticles have been proposed as nanocarrier of triarylmethyl oxygen spin probes for *in vivo* oxygen mapping using electron paramagnetic resonance (EPR) imaging. However, the weak spectral dependence of these probes on mobility makes it more challenging to study the radical location and mobility when mixed with MSNs. We used ^13^C_1_ isotopologues of two popular oxygen EPR spin probes (dFT and Ox071) highly sensitive to molecular tumbling to study the effect of the probe loading into MSNs on probe mobility. We found that when using homemade or commercially available cationic MSNs, the loading of both probes inside MSNs leads to a drastic decrease in the tumbling rate and strong dipolar broadening induced by the high local probe concentration inside the MSNs. In the case of ^13^C_1_-Ox071, a small fraction of the probe (∼15%) in interaction with the MSNs can tumble at a higher rate but five times slower than without MSNs. For both probes, the immobilization and self-relaxation inside MSNs hamper the oxygen-sensing properties of the nonlabeled probes (Ox071 or dFT) used for EPR oximetry. Free tumbling and the absence of self-broadening have been reported for Ox071 inside cationic MSNs.^29^ However, in our hands, homemade or purchased cationic MSNs lead to the immobilization of the probe inside the nanoparticles and significant self-broadening, even at a 50-fold lower concentration than the one reported.^29^ Therefore, it is critical to characterize the effect of the loading of the probe inside MSNs when using TAM-loaded MSN probes for oxygen imaging, as the manufacturing of MSNs may significantly affect the spectral properties of the probes inside the nanoparticles. The pH of the solution is also expected to have a significant role in the loading, mobility, and release of the probes from the MSNs. Other parameters, such as the ionic strength and temperature, are also expected to have an effect. However, the full studies of the influence of those parameters fall outside the scope of this report, which aims to raise attention to the challenges when using TAM-loaded MSNs for EPR oximetric applications.

## Supporting Information Available

Zeta potential titration of hMSNs and TEM images (PDF)
