# Supplementary material for: Evaluation of Mesoporous Silica Nanoparticles as Carriers of Triarylmethyl Radical Spin Probes for EPR Oximetry
Source: J Phys Chem B. 2025 Jan 30;129(6):1846–54. doi: 10.1021/acs.jpcb.4c06480 (PMC11825265; doi:10.1021/acs.jpcb.4c06480)
Supplement: Supplementary file 1 — jp4c06480_si_001.pdf [file jp4c06480_si_001.pdf]

Supporting Information

Evaluation of Mesoporous Silica Nanoparticles as Carrier of Triarylmethyl Radical Spin Probes for EPR Oximetry

Misa A. Shaw[a,b,c], Martin Poncelet[a,b], Derrick A. Banerjee[d], Konstantinos A. Sierros[d], and Benoit Driesschaert[a,b,c,e*]

[a] Department of Pharmaceutical Sciences, School of Pharmacy, West Virginia University, Morgantown, WV, 26506, USA

[b] In Vivo Multifunctional Magnetic Resonance Center, Robert C. Byrd Health Science Center, West Virginia University, Morgantown, WV, 26506, USA

[c] West Virginia Clinical and Translational Science Institute, West Virginia University, Morgantown, WV, 26506, USA

[d] Department of Mechanical, Materials and Aerospace Engineering, Benjamin M. Statler College of Engineering and Mineral Resources, West Virginia University, Morgantown, WV 26506, USA

[e] C. Eugene Bennett Department of Chemistry, West Virginia University, Morgantown, WV 26506, USA

Email for the corresponding author: benoit.driesschaert@hsc.wvu.edu

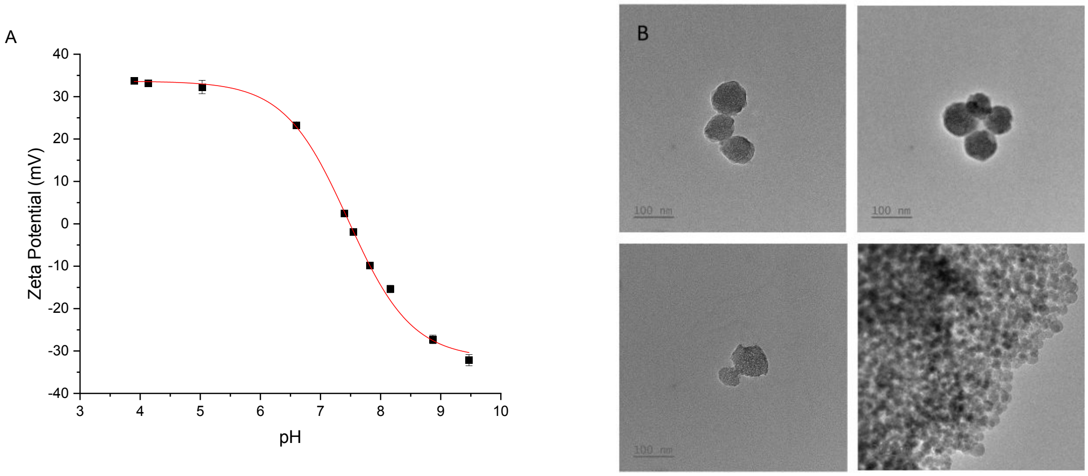

Figure S1. (A) Zeta potential titration of hMSN, 6 mg of MSNs in 3 mL of DI water was titrated with small amounts of NaOH or HCl (< 3% dilution)**. (B)** TEM images of hMSN with enlarged pore size.
